# Assessment of body composition in obese patients undergoing one anastomosis gastric bypass: cross-sectional study

**DOI:** 10.1038/s41598-020-75589-2

**Published:** 2020-11-03

**Authors:** Jose-Maria Jimenez, Jaime Ruiz-Tovar, María López, Artur Marc-Hernandez, Miguel-Angel Carbajo, Maria-Jose Cao, Sara Garcia, Maria-Jose Castro

**Affiliations:** 1 Nursing Faculty, University of Valladolid, Valladolid, Spain; 2 Centre of Excellence for the Study and Treatment of Obesity and Diabetes, Valladolid, Spain; 3 Laboratory of Training Analysis and Optimization, Sports Research Center, Miguel Hernández University, Elche, Alicante, Spain; 4 Department of Anatomy and Surgery, Alfonso X University, Madrid, Spain

**Keywords:** Health care, Endocrinology, Obesity

## Abstract

Bariatric surgery is the most effective long-term treatment to obesity, and it is necessary to assess changes in body composition and to be able to establish better follow-up of patients. Cross-sectional, observational study in patients undergoing One Anastomosis Gastric Bypass (OAGB) bariatric surgery. We analysed changes in weight and body composition during the first postoperative year. 405 patients (68.9% women. 31.1% men), mean age 44 years, mean weight 110.02 kg, Body Mass Index (BMI) 39.76 kg/m^2^, height 1.66 m. The variables analyzed were substantially decreased compared to the preoperative values one year after surgery in every case: weight (110.02 ± 22.03 kg vs. 69.36 ± 13.60 kg), BMI (39.76 ± 6.65 vs. 24.52 ± 3. 76), fat free mass (61.12 ± 12.43 kg vs. 53.61 ± 11.61 kg), fat mass (50.44 ± 14.36 kg vs. 15.74 ± 6.74 kg), bone mass (58.06 ± 11.85 kg vs. 50.92 ± 11.06 kg) and water (45.08 ± 9.99 kg vs. 37.39 ± 9.23 kg), P < 0.001. The results show noticeable improvements in weight reduction and changes in body composition, and will contribute to develop a thorough understanding of both of them, contributing also to perform a better patients’ follow-up.

## Introduction

Obesity has become a public health challenge^1^. It is an epidemic that has tripled worldwide since 1975; 39% of adults are overweight (39% of men and 40% of women) and 13% are obese (11% of men and 15% of women)^2^.

It is a health issue with adverse clinical consequences in almost every system of the body; it leads to psychological problems and has a major economic impact on national health systems^1,3,4^.

Nowadays, bariatric surgery is considered the most effective treatment in long-term control of obesity and its main associated comorbidities^5^. There are different surgical techniques of restrictive and malabsorptive type, in which the criteria of choice depend on each patient and surgical team. The International Federation for the Surgery of Obesity and Metabolic Disorders (IFSO), has described bariatric surgeries worldwide in order to establish in the future a profile of patients undergoing surgery^6^.

One Anastomosis Gastric Bypass (OAGB), is one of the bariatric surgeries of malabortive type accepted by IFSO with effective long-term results in weight loss and associated comorbidities^7^. The results in weight reduction can be interpreted in absolute terms as weight and Body Mass Index (BMI)^8^. Other internationally accepted relative terms to describe success in weight loss after surgery are the percentage of Excess of BMI Lost (%EBMIL), which defines the success of a surgery in values greater than 50%^8,9^.

Changes in body composition determined by fat mass (FM) and fat-free mass (FFM) composition should become a standard outcome measure used to assess postoperative weight loss^10^. The assessment of postoperative weight loss is limited to weight change outcomes, not including body composition variations, which would facilitate better assessment of results and clinical management of patients^10,11^.

Several international organisations involved in the study of obesity acknowledge the need to use, in a consensus basis, tools to assess nutritional status that allow an improvement in the treatment and medical nutritional support, having a great potential to reduce the morbidity burden of the obese patient population^12^.

The success of surgery is not only based on reaching normal weight; bariatric surgery teams must maintain regular multidisciplinary follow-ups of patients, assessing nutritional aspects and emotional support in this important life change^13,14^. It is therefore necessary to consider possible malnutrition causes related to surgery and to be able to establish appropriate therapeutic guidelines and nutritional supplementation to correct them^13^.

The assessment of body composition after bariatric surgery has still room for improvement in the follow-up and in most reported cases the information is not complete. Thus, this study aims to evaluate changes in weight and body composition after 6 and 12 months in patients who underwent OAGB surgery.

## Methods

### Study design and sample

The study was cross-sectional and observational, and it was conducted in 405 patients diagnosed with obesity who underwent the bariatric surgery of malabsorptive type ‘One Anastomosis Gastric Bypass (OAGB)’ between January 2015 and July 2018 at an IFSO accredited Center of Excellence.

### Data collection

The preoperative protocol was conducted by a multidisciplinary team that approached prospective bariatric surgery patients. It included nutritional status assessment to determine dietary habits as well as complementary weight and anthropometry variables, psychological evaluation, analysis of cardiorespiratory function, and other complementary studies of associated comorbidities if necessary.

The inclusion criteria for patients followed the recommendations approved by the International Federation for the Surgery of Obesity and Metabolic Disorders (IFSO): BMI > 40 kg/m^2^, or BMI > 30–35 kg/m^2^ with existing comorbidities linked to obesity and poor metabolic control, of legal age, and willing to participate in the study by giving their written consent.

OAGB is a malabsorptive surgical procedure with laparoscopic access, conducted by the same surgical team under general anesthesia, in which it is performed a termino-lateral gastrojejunal anastomosis and the creation of an estimated gastric reservoir of an approximate volume of 30 ml^7^.

The assessment of body composition and anthropometry was conducted in the preoperative consultation and in the subsequent 6 and 12-month follow-ups after bariatric surgery. The variables analysed were: age, gender, height, weight, BMI [weight (kg)/height^2^(m)]. The criteria considered to evaluate weight loss were: percentage of excess body mass index loss (%EBMIL) and percentage of total weight loss (%TWL):

- %EBMIL = [(Preoperative BMI − current BMI)/(preoperative BMI − 25)] × 100
- %TWL = (Current weight/preoperative weight) × 100

On the other hand, the variables to assess body composition were: fat-free mass (FFM), fat mass (FM), bone mass (BM), muscle mass (MM) and water were measured by the TANITA BC-420MA body composition analyser. The TANITA BC-420MA is a body analyser with four stainless steel electrodes located on the lower platform for body compartment analysis. The patient is placed on them in bare feet and the results are determined within 15 s.

The accuracy of bioimpedance analysis has been validated in bariatric patients by several studies^15^.

### Ethical considerations

All participants agreed to the conditions of participation in the study and the use of their data was anonymous and confidential. The study was approved by the Ethics Committee of the Nursing Faculty of the University of Valladolid (identification code: 2019/JMJP71), following the protocols of the latest update of the Declaration of Helsinki. We obtained written consent from all subjects of the study.

### Statistical analysis

The data gathered were analysed using IBM SPSS Statistics v. 25.0 software. The quantitative variables that followed a normal distribution were summarized as means ± standard deviations (SD), and medians and ranges were recorded for non-Gaussian variables. Qualitative variables were summarized by number and as percentage of cases. The paired t test or Wilcoxon rank test was used to study the differences between means over time, and ANOVA or the Kruskal–Wallis test for comparing changes in variables with more than two categories. Lastly, statistical significance was set at P < 0.05.

## Results

The sample is constituted by 405 patients, 279 women and 126 men, age 44.39 ± 11.49 years. In the preoperative examination, the average weight was 110.02 ± 22.03 kg, BMI 39.76 ± 6.65 kg/m^2^, and height 1.66 ± 0.08 m.

The body composition variables analysed before surgery were: FFM = 61.12 ± 12.43 kg; FM = 50.44 ± 14.36 kg; BM = 3.06 ± 0.57 kg; MM = 58.06 ± 11.85 kg; Water = 45.08 ± 9.99 kg. Table 1 depicts the general characteristics along with the preoperative body composition variables of the sample divided by gender.

**Table 1:** General characteristics and body composition of patients before bariatric surgery.

	Women (n = 279)	Men (n = 126)	P-value
Age (years)	44.08 ± 11.96	45.07 ± 10.38	0.33
Height (m)	1.62 ± 0.06	1.74 ± 0.06	< 0.001
Weight (kg)	104.85 ± 17.78	124.23 ± 21.94	< 0.001
BMI (kg/m^2^)	39.48 ± 6.99	40.27 ± 5.98	0.24
FFM (kg)	54.71 ± 6.96	73.14 ± 11.49	< 0.001
FFM (%)	52.74 ± 5.15	59.65 ± 8.33	< 0.001
FM (kg)	50.13 ± 12.37	50.99 ± 17.49	0.92
FM (%)	47.26 ± 5.15	40.34 ± 8.33	< 0.001
BM (kg)	2.77 ± 0.34	3.59 ± 0.53	< 0.001
MM (kg)	51.94 ± 6.62	69.53 ± 10.96	< 0.001
Water (kg)	39.65 ± 5.56	55 ± 8.57	< 0.001

Data are mean ± SD.

BMI: Body Mass Index; FFM: Fat Free Mass; FM: Fat Mass; BM: Bone Mass; MM: Muscular Mass.

Although the mean initial weight was higher in men, the mean FM was homogeneous among women and men (50.13 ± 12.37 kg vs. 50.99 ± 17.49 kg respectively). FFM was higher in men (73.14 ± 11.49 kg vs. 54.71 ± 6.96 kg), as well as MM (69.53 ± 10.96 kg vs. 51.94 ± 6.62 kg), and water (55 ± 8.57 kg vs. 39.65 ± 5.56 kg) P < 0.001. Initial bone mass was significantly lower in women (2.77 ± 0.34 kg vs. 3.59 ± 0.53 kg) P < 0.001.

The evolution of weight loss and variation of body composition (Table 2) reveals the highest weight loss at 12 months after surgery. The average weight was 69.36 ± 13.60 kg. Weight loss is positively correlated with the reduction of BMI in postoperative controls after 6 months (r = 0.764. P < 0.001) and after 12 months (r = 0.830. P < 0.001).

**Table 2:** Evolution of weight loss and postoperative body composition.

	Preoperative	6 months	12 months
Weight (kg)	110.02 ± 22.03	72.46 ± 11.26*	69.36 ± 13.60*
BMI (kg/m^2^)	39.76 ± 6.65	26.50 ± 3.84*	24.52 ± 3.76*
EBMIL (%)	–	97.98 ± 29.59	119.96 ± 35.47**
TWL (%)	–	36.12 ± 5.20	38.79 ± 7.68**
FFM (kg)	61.12 ± 12.43	52.61 ± 8.69*	53.61 ± 11.61*
FFM (%)	55.19 ± 7.24	73.07 ± 8.90*	77.45 ± 7.97*
FM (kg)	50.44 ± 14.36	19.84 ± 8.42*	15.74 ± 6.74*
FM (%)	44.81 ± 7.24	26.92 ± 8.90*	22.54 ± 7.97*
BM (kg)	3.06 ± 0.57	2.65 ± 0.40*	2.69 ± 0.54*
MM (kg)	58.06 ± 11.85	49.95 ± 8.28*	50.92 ± 11.06*
Water (kg)	45.08 ± 9.99	37.15 ± 6.39*	37.39 ± 9.23*

Data are mean ± SD.

BMI: Body Mass Index; FFM: Fat Free Mass; FM: Fat Mass; BM: Bonne Mass; MM: Muscular Mass.

*Significant difference from preoperative values, P < 0.001.

**Significant difference from 6 months follow-up, P < 0.001.

All body composition variables were significantly reduced after surgery, as shown in Table 2. (P < 0.001). The ratio of FFM to FM correlate negatively after 6 and 12 months post-surgery respectively (r = − 1.000. P < 0.001), thus increasing the FFM percentage and decreasing the FM percentage in the postoperative period up to 12 months.

**Figure 1:**
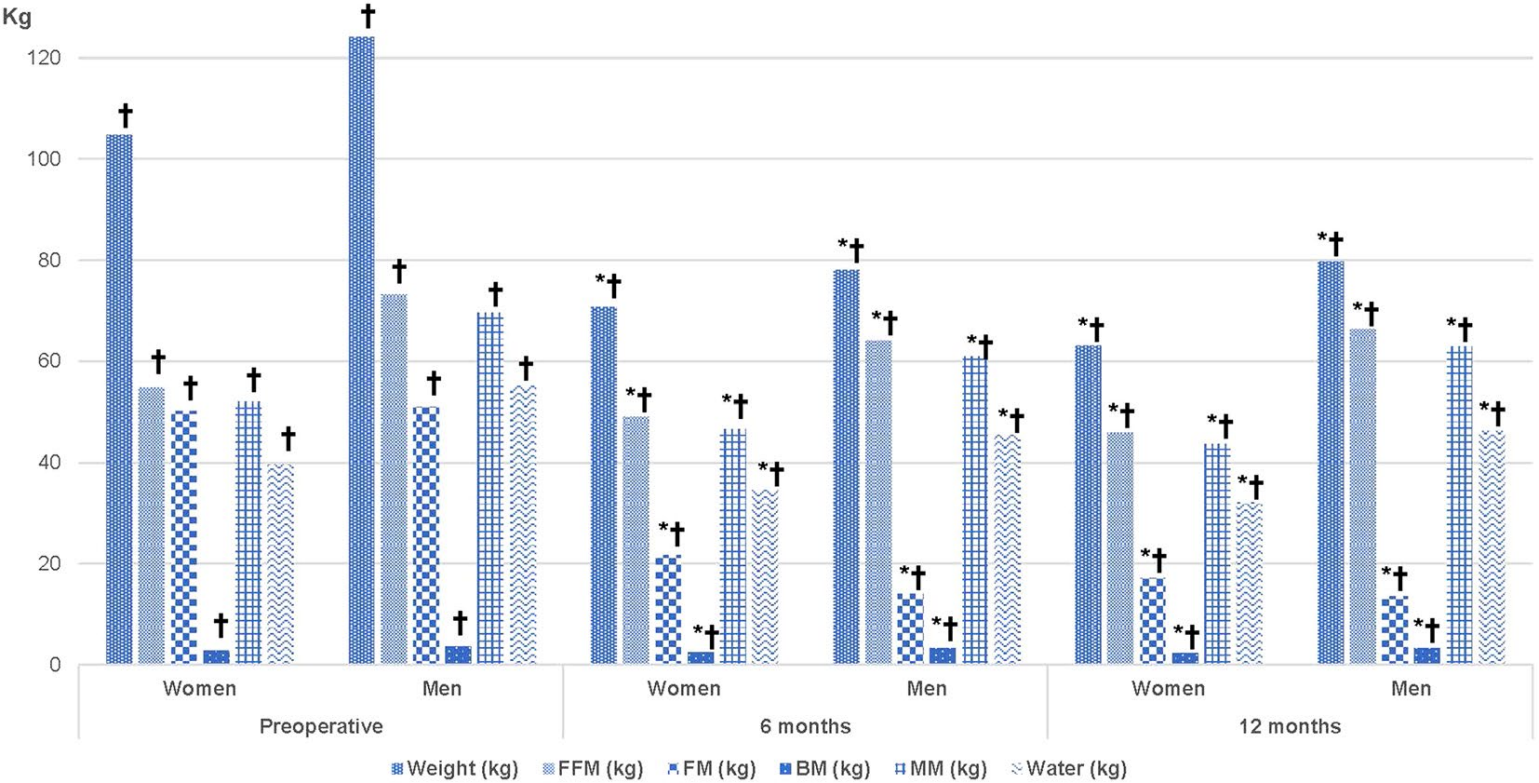
Assessment of the evolution of weight loss and body composition by gender. Data are mean ± SD. BMI: Body Mass Index; FFM: Fat Free Mass; FM: Fat Mass; BM: Bone Mass; MM: Muscular Mass. *Significant difference from preoperative values, P < 0.05. ^†^Significant difference between sex, P < 0.05.

It is worth noting the significant correlation of postoperative weight loss to BM reduction at 6 months (r = 0.675. P < 0.001) and at 12 months (r = 0.875. P < 0.001), although the initial BM value in women was significantly lower than in men. Figure 1 depicts a significant reduction in BM from the preoperative stage to 12 months postoperative in both women (2.77 ± 0.34 kg vs. 2.33 ± 0.22 kg) and men (3.59 ± 0.53 kg vs. 3.28 ± 0.38 kg) P < 0.001.

When assessing the evolution of weight loss and body composition by gender (Fig. 1), there was a significant difference in every variable with the exception of preoperative FM, and the %EBMIL and %TWL at 6 and 12 months. FFM decreased in both gender groups after surgery, although the proportion was always higher in men; the same applies to BM and MM. Although FM in both genders is lower after surgery, it is noted that women have higher mean values than men at the 6 and 12-month postoperative follow-ups.

**Figure 2:**
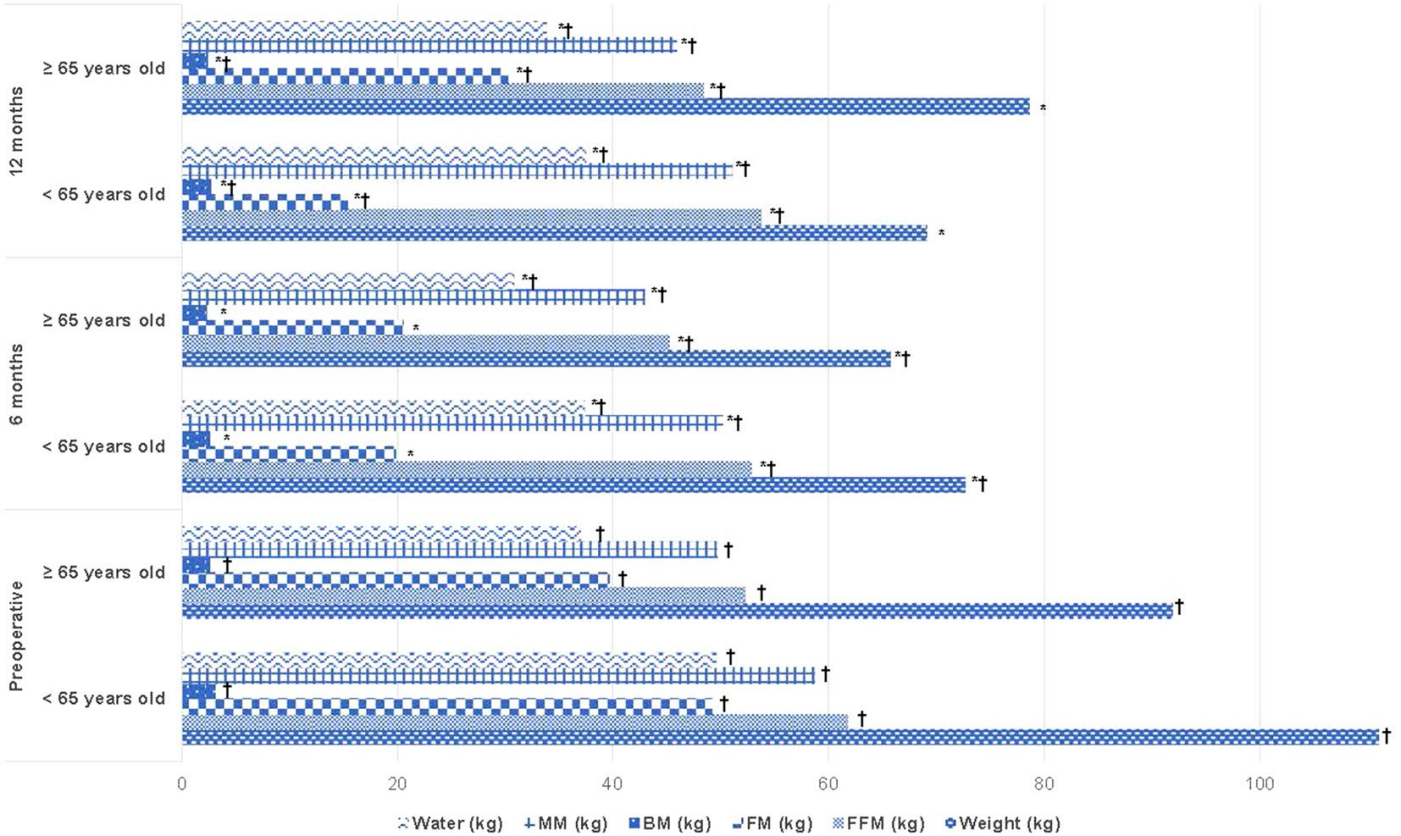
Assessment of weight loss and body composition by age group. Data are mean ± SD. BMI: Body Mass Index; FFM: Fat Free Mass; FM: Fat Mass; BM: Bone Mass; MM: Muscular Mass. *Significant difference from preoperative values, P < 0.05. ^†^Significant difference between age group, P < 0.05.

The sample was divided into two age groups, < 65 years old and ≥ 65 years old, to describe the evolution of the postoperative body composition. 383 patients were < 65 years old (94.5%) and 22 patients ≥ 65 years old (5.5%). The initial weight was significantly higher in the < 65 years group compared to the ≥ 65 years one (111.03 ± 21.78 kg vs. 91.93 ± 20.02 kg) P < 0.05. Figure 2 shows the postoperative weight and body composition changes.

In both age groups, a significant weight reduction occurred after surgery, with the lowest midpoint of weight reached by the < 65 years group at 12 months (69.12 ± 13.69 kg) as opposed to the ≥ 65 years group, that reached it at 6 months (65.70 ± 9.84 kg). The evolution of the analysed body composition variables follows the same trend as the general sample studied. There were significant differences in the BM from the preoperative moment, in which the younger group had a higher average value, although in both the reduction of BM was substantial up to the 12-months follow-up (Fig. 2).

## Discussion

OAGB bariatric surgery contributes to effective weight reduction and has a large impact on the change of body composition variables in the assessments both after 6 and 12 months. Not all bariatric surgeries achieve the same postoperative results in weight control: the most effective is the malabsorptive type^3,5,7^. The results reported at the 12-month follow-up in malabsorptive surgeries such as the Roux-en-Y gastric bypass (RYGB) reach mean weight values of 83.4 ± 13.6 kg^16^, as opposed to restrictive surgeries such as the sleeve gastrectomy (SG) with 81.5 ± 15.6 kg^17^, even though the preoperative weight in the RYGB is higher than that of the SG (124.8 ± 25.6 kg vs. 120.9 ± 19.7 kg)^16,17^.

In our study the average weight after 12 months was lower (69.36 ± 13.60 kg), although it should be noted that the average preoperative weight of the sample was lower than in other studies (110.02 ± 22.03 kg).

Bhandari et al.^18^ reviewed the results of %TWL, which were significantly higher in OAGB after 6 months (28.75 ± 5.23%) compared to RYGB (21.54 ± 5.97%. P < 0.001). In our study, %TWL at 6 and 12 months after surgery was higher (36.12 ± 5.20%. 38.79 ± 7.68% respectively).

Other studies that start with a higher average preoperative BMI than our sample (42.7 ± 5.2 kg vs. 39.76 ± 6.65 kg), achieve lower %EBMIL both at 6 months (61.3 ± 21.9% vs. 97.98 ± 29.59%) and at 12 months after surgery (78.3 ± 25.7% vs. 119.96 ± 35.47%)^19^.

In our study, we observed an average weight reduction at 12 months after surgery greater in men than in women (46.30 ± 17.75 kg vs. 41.01 ± 9.96 kg), as well as in other studies that show reductions of 36.5 ± 7.0 kg in men and 33.6 ± 8.4 kg in women^16^. However, the initial weight of our study was higher in men, as in most other works^16,20,21^. In contrast, %TWL at 12 months after surgery was higher in women than in men (40.35 ± 6.31 kg vs. 36.62 ± 9.07 kg), following the same trend found in other studies (28.8 ± 8.6 kg vs. 28.2 ± 8.3 kg)^21^.

There are very few studies that analyse changes in body composition after bariatric surgery. Nevertheless, we can compare the results obtained from OAGB with the RYGB malabortive technique because it is the most similar technique in terms of results on weight control and associated comorbidities^3–5^.

As well as to weight reduction, the impact on changes in body composition is more evident in malabsorptive than restrictive surgeries, although few studies analyse changes after OAGB surgery^10^.

Davidson et al.^22^ compare the changes after RYGB, SG and adjustable gastric band (BAND) obtaining the lowest mean weight results at 12 months after surgery, as in our study, in RYGB and SG (79.8 ± 16.3 kg vs. 104.3 ± 25.2 kg, respectively). FFM before RYGB was higher in men than in women (57.6 ± 8.4 kg vs. 74.9 ± 9.3 kg); these values were similar to those described in our study (54.71 ± 6.96 kg vs. 73.14 ± 11.49 kg). The mean values of FFM after a year of RYGB and OAGB in women (50.4 ± 7.1 kg vs. 46.03 ± 4.65 kg) and men (46.03 ± 4.65 kg vs. 66.25 ± 8.14 kg) followed the same trend, considering that in the study by Davidson et al*.* the mean preoperative weight was lower than in the OAGB, both in women and men (123.1 ± 18.9 kg vs. 143.8 ± 17.2 kg)^22^.

As in Alba et al.’s study^23^, in which men had a higher FFM than women before surgery, they lost a higher FFM compared to women 6 and 12 months after surgery (12 ± 4 kg vs. 8 ± 4 kg at 12 months, respectively; P = 0.01).

Although our results followed the same trend, there was a greater loss of FFM in both gender groups compared to the study by Alba et al.^23^ after 6 months (6.05 ± 3.93 kg vs. 7.85 ± 6.50 kg, women vs. men; P < 0.05) and after 12 months (7.63 ± 2 kg vs. 8.22 ± 7.32 kg, women vs. men; P < 0.05).

Similar to this work, most authors describe a proportionally greater decrease in postoperative FM than the loss of FFM^17,24,25^. The correlation between weight loss and body composition parameters was substantial, regardless of the mean preoperative weight^17^.

It is essential to assess the loss of muscle and bone mass, especially due to the clinical consequences and the impact in the management of postoperative nutrient deficiencies. In all the studies analysed the loss of MM is substantial^15–17,20–22^, revealing a similar trend by gender one year after surgery in women and men (5.9 kg vs. 8 kg)^22^, OAGB (7.24 ± 1.92 vs. 7.83 ± 6.98 kg).

In our study, mean BM values at 12 months after surgery were 2.69 ± 0.54 kg, similar to those obtained in other researches 3.0 ± 0.6 kg^16^, 2.6 ± 0.4 kg^26^. Although the decrease of BM at 12 months after surgery in women versus men was very similar (0.38 ± 0.09 vs. 0.39 ± 0.34 kg), it is necessary to consider that preoperative BM was significantly lower in women (2.77 ± 0.34 kg vs. 3.59 ± 0.53 kg; P < 0.05).

Few authors have described postoperative results in ≥ 65-year-old patients. Our study sample demonstrates an effective weight loss control, comparable to < 65 years-old patients^27^, obtaining the younger group better results in weight control and changes in body composition^28^.

These changes in body composition are linked to a great impact on weight reduction, especially during the first postoperative year^7–9,17,21^. It is at this point when an optimal personalized control of each patient should be emphasized to prevent nutrient deficiencies and improve postoperative supplementation^17,21,26^.

Among the limitations of this work is the lack of randomness of the analysed sample, as well as short-term results. It should be completed with the supervision of long-term results and comparisons with other bariatric surgeries. However, these are assumable limitations and allow to accomplish the purpose of the study.

## Conclusion

The changes in weight reduction and body composition of OAGB patients during the first postoperative year were statistically significant, showing effective results in obesity control.

Variations in body composition were correlated with changes in weight. Variations in FFM, FM, MM, BM were similar in both genders, with the exception of the modification of FM after 6 months, which was greater in women. The percentage of FM was always significantly higher in women.

The evolution of weight and body composition after surgery of ≥ 65-year-old patients, maintained the same trend as the < 65-year-old group, although the changes were more substantial in the younger group.

The implementation of the results obtained on the changes in weight and body composition after bariatric surgery will enable better follow-up of patients and more comprehensive information on weight reduction and changes in body composition after surgery.
